# A crisis resolution and home treatment team in Norway: a longitudinal survey study Part 1. Patient characteristics at admission and referral

**DOI:** 10.1186/1752-4458-6-18

**Published:** 2012-09-19

**Authors:** Ottar Ness, Bengt Karlsson, Marit Borg, Stian Biong, Suzie Kim Hesook

**Affiliations:** 1 Faculty of Health Sciences, Buskerud University College, Box 7053, 3007, Drammen, Norway

**Keywords:** Crisis resolution, Mental health home treatment, Mental health services, Community mental health

## Abstract

**Background:**

Crisis resolution and home treatment (CRHT) is an emerging mode of delivering acute mental health care in the community. There is a paucity of knowledge regarding the workings of CRHT in the literature. This is the first paper in a series of three from the longitudinal survey of patients of a CRHT team in Norway, which was aimed at describing the characteristics of patients served, professional services provided, and clinical outcomes. This report focuses on describing the characteristics of the patients at admission.

**Methods:**

The study was a descriptive, quantitative study based on the patient data from a longitudinal survey of one CRHT team in Norway. The participants of the survey, a total of 363 patients, were the complete registration of patients of this team in the period from February 2008 to July 2009.

**Results:**

Although diverse in their characteristics, the patients were over represented by females, young to middle aged, and people on public support. The patients were mostly referred to the team by self/family members and primary care physicians. At admission, depression was the most prevalent symptom, the overall intensity level of mental health problems was low, and most of the patients had long-standing mental health problems.

**Conclusions:**

Self/family referral seems to be a critical route to receive services by CRTH teams as shown in our study, suggesting a need to examine policies that disallow this form of referral in some communities. The findings from our study show that the patients of the CRHT team, while mostly having long-standing mental health problems and had been receiving healthcare for them, did not have severe mental health problems at admission, although could have been in crises. There is a need for further studies to examine how people with severe mental health problems obtain services in time of crises, and to address the need to gain a greater understanding of the role of CRHT.

## Introduction and background

This is the first in a series of three reports presenting findings from a longitudinal survey of a crisis resolution home treatment (CRHT) team in Norway [1,2]. This study was based on the assessment, treatment, and outcome registration-data of a total of 363 patients of the CRHT team in a period from February 2008 to July 2009. The focus of this article is on patients’ characteristics at admission.

During the last four decades comprehensive changes have been seen in the mental health service systems with the intention to benefit patients and their families and in response to the need to streamline healthcare systems for economic reasons [3,4]. Community care models are being established in order to minimize hospitalization and extend acute care and rehabilitation within the context of family and immediate social environment of individuals [5,6]. One model that has been developed in response to this trend is crisis resolution home treatment team (CRHT). A CRHT team is a type of the so-called ‘functional teams’ developed in the United Kingdom as part of the National Service Framework [2,7]. In line with the emphasis on community based treatment and rehabilitation for mental health care by the World Health Organization [8] and the European policies, the overall objective of these teams is to offer comprehensive treatment and support in people’s home environment and prevent hospital admission. CRHT teams aim to provide an alternative to hospital admission, robust psychosocial as well as psychiatric assessments, gate-keeping of hospitalization, and opportunities to resolve crisis in the contexts of their occurrence [2,7,9,10].

In Norway CRHT was introduced as a part of the National Action Plan for Mental Health in 2005 [11,12]. A national strategy was formulated in Norway to establish a CRHT team at each of the 78 community mental health centers (DPS) by the end of 2009 with an overall objective to offer comprehensive mental health treatment and support in people’s home environment and prevent hospital admission. A set of guidelines was established based on international experiences with the key service characteristics being defined as (a) brief responding time, (b) provision of assessment and direct care in the context of home and family, (c) working in partnerships with relevant health and social welfare providers, and (d) assessment and course of action that may include inpatient treatment, home treatment, crisis resolution by the team, and next-level referrals to health and social services [12]. CRHT teams, as their role is to respond to mental health crises in the community, do not have a role in the care of patients in acute hospitalization, having no direct role in determining the length of their hospital stay specifically. However, there has been an implication in the literature that the availability of CRHT teams in communities and the care by CRHT teams may influence hospitalization rates and the length of hospital stay [13,14].

A literature review by Sjølie *et al.*[4] revealed that most of the published articles on CRHT teams focus on structural issues pertaining to the development of home treatment services and on macro-level outcomes such as cost-effectiveness and admission rates, which have political, economic, and practical implications. There has been less attention in the literature describing patients of CRHT teams, referral procedures, and status of mental health at admission, which will be the focus of this article. Hasselberg, Gråwe, Johnson and Ruud [15] described in their study of eight crisis resolution teams in Norway that the socio-demographic and clinical characteristics of the patients were that: (a) the users were mostly aged between 20-50 years, (b) a little more than half of the patients were women, (c) approximately half of the patients were unmarried and living alone, (d) one-quarter were in paid employment, (e) 99% were Norwegian in their ethnicity, compared to 92% of the general population of Norway, (f) the majority of the patients had primarily mood and anxiety disorders, and 14% had psychotic symptoms, (g) about 60% had previous contacts with mental health services, (h) 38% of the patients had received treatment at an outpatient unit within the past 12 months and 22% had been in an inpatient ward, and (i) three in four were emergency referrals and 25% had self-referred to the CRHT teams.

The aims of this article were to explore and describe (a) the basic characteristics of the patients of one CRHT team in a health region in Norway, (b) the referral sources, and (c) the status of mental health at the time of admission. The ultimate purpose was to gain an in-depth understanding about patients of CRHT teams with the research questions: What are the characteristics of patients, how do they seek the services of CRHT teams, and what are their major problem areas in seeking the services of CRHT teams?

## Methods

### Design

The study was a descriptive, quantitative study based on the patient data from a longitudinal survey of one CRHT team in Norway. The study was conducted by following a CRHT team that was established in September 2007 for a period of 18 months from February 2008 to July 2009.

### Participants

The CRHT team is located in an area of five municipalities spread out in an urban and rural district in the southeast region of Norway, with a population of 130,000 inhabitants. The participants of the survey, a total of 363 patients, were the complete registration of patients of this team in the period from February 2008 to July 2009.

### Description of the CRHT team and the general protocols for service

CRHT teams in Norway were proposed to increase accessibility to specialized mental health services for patients experiencing acute mental health crisis [15]. The teams were to offer rapid assessment with 24/7 availability, and provide an alternative mode of treatment to hospitalization [15]. The Norwegian mental health system for adults consists of three service levels: (a) at the first level there are primary care physicians and mental health professionals as individual practitioners or teams in primary care settings, (b) at the second level there are community mental health centers of District Psychiatric Service (DPS) for a pre-determined catchment area, which organize service units of outpatient clinic and services, day-care centres and services, and functional community mental health care teams such as CRHT teams, drug/alcohol abuse teams, psychosis/rehabilitation/ambulatory teams, and day/group teams, and (c) at the third level, there are psychiatric hospital wards, including acute wards for in-patient services [15]. People in the community may receive mental health services from private psychiatric mental health professionals in practice in the community, go to outpatient clinics, attend day-care centres, or receive services from various functional teams. In each DPS, there are acute hospital beds designated as crisis beds, admission unit beds, open-unit beds, and closed-unit beds. The specific characteristics of CRHT teams are that they are to aim for the resolution of mental health crises in the community, provide services at patients' homes, respond to patients within a 24 h period, are organized as multidisciplinary teams, and determine whether or not patients admitted to the team need to be hospitalized. There is no specific guideline regarding the response time to referrers. However, since responses to patients are expected to be carried out within 24 h, the expectation is that responses to referrers, especially to non-self referrals, to be within a few hours of initial contacts. CRHT teams have been developed to prevent hospitalization of patients who could otherwise be successfully helped in the community by the team. However, CRHT teams do not have the gate-keeping authority to make hospitalization decisions for all inpatient admissions in communities, only for those who are admitted to the teams.

The CRHT team studied in this research project was established in September 2007 for this district in response to the national mandate for the establishment of a CRHT team in each of the 78 DPS in Norway, and was one of the earliest teams that were established. This CRHT team had 12 therapists, including the managing director. The team included one psychologist, nine nurses and two social workers, who were all prepared to postgraduate level in either psychiatric nursing or mental health work. In addition, one psychiatrist from the DPS worked with the team on a part time basis providing medical services. There was no staff turnover during the study period. The team was in operation at both daytime and evening hours during the week and only daytime on weekends. The staffing level at the time only permitted the team to operate from 8 am to 10 pm on weekdays and from 8 am to 3:30 pm on weekends. During the opening hours healthcare professionals, patients, family members, and friends were able to make calls directly to the CRHT team for referral. Thus, the team was not available 24/7, and did not function formally as the gate keeping unit for psychiatric hospitalizations in the DPS.

The community mental health services of this DPS were organized in the same way as the general configuration for all DPS in Norway. Neither the data on psychiatric morbidity nor admission diagnoses of psychiatric admission are available for the DPS; however there were a total of 42 acute psychiatric in-patient beds for the DPS at the hospital: 1 DPS bed designated as the crisis bed; four acute wards - the admission ward with 6 beds, one open ward with 15 beds, and two closed wards with 10 beds each. Although there were some variations in the ways patients were processed for services by the team, the team followed the general protocol as outlined below:

1. Referral phone call is received from a patient, family member or professional such as primary care physician, private psychiatrist, or nurse.

2. The referral telephone call is screened by the person regarding the appropriateness for admission to the CRHT service, and the screening is discussed and evaluated by the team.

3. As the call is determined to be appropriate for the team’s service, a team member creates a clinical record for this patient to begin the admission process.

4. A team meeting is held to assign a team member to this patient.

5. The assigned team member meets with the patient (usually at the patient's residence) in order to assess the crisis situation, fill out the admission registration form that includes an initial assessment, and to decide on intervention plans and further contacts with the patient.

6. The assigned team member continues with the established service plan for the patient.

7. A team meeting is held to decide on a discharge plan.

8. The assigned team member meets with the patient to complete the discharge data form.

9. The team can make decisions regarding hospitalization of patients anytime after their admission to the team. Hospitalization would be one of the discharge destinations for patients.

Therefore, the data for this study were from the patients who were admitted to the CRHT team. A finding from another data set regarding the total number of referral calls received by this team during 18 months from May 2008 to December 2009 was 1,117 of which 418 patients were admitted to the team. We estimate that a similar number of referral calls would have been received by the team during our study period, suggesting that about one third of the referral calls were admitted to the team. There were no data except the basic demographic information on those individuals who were referred but not admitted to the CRHT team. This means that there were no data on the exact nature of communication at the time specifically regarding the reasons for not admitting the patients. However our knowledge of the team suggests that they would have been told to seek other appropriate services in the community such as clinics or day-care centres. Referrals to inpatient psychiatric emergency units would have been done after initial assessments.

### Instruments

A registration form was used to collect the data, and was based on the Multicentre Study on Acute Psychiatry (MAP) [15]. This data form was used to register the CRHT service as a part of a larger study, which included an aggregated data on five CRHT teams in Norway from which a report has been made [2] as well as the patient registration data used in this study. This data set will also be used to report the service processes and outcomes in two reports planned in a series including this report. The data set for this study addressed the team's actual service in terms of referrals and sources of referrals, patients' personal background, service duration, services provided, and discharge destination. The unit of the registration was patient for our study, with the data obtained at intake and discharge. The data form consisted of eight sections of which we are reporting on the data from the first four sections only in this paper: (a) intake information including referral sources, (b) personal background information, (c) services received prior to the intake, (d) intake assessment, (e) services provided by the team, (f) types of coordination and cooperation contacts made by the team, (g) discharge assessment, and (h) discharge follow-up recommendations. For assessments of patients' mental health status both at intake and discharge, the Health of the Nation Outcome Scale (HoNOS) [16,17] was used. The HoNOS instrument measures severity of mental health problems in the following 12 categories:

1. Overactive, aggressive, disruptive or agitated behavior

2. Non-accidental self-injury

3. Problems with alcohol or substance abuse

4. Cognitive problems

5. Physical illness or disability problems

6. Problems associated with hallucinations and delusions

7. Problems with depressed mood

8. Other mental and behavioral problems, including ten items (*a = phobia, b = anxiety, c = compulsive behaviors, d = stress/tension, e = dissociative, f = somatoform, g = eating disorder, h = insomnia, i = sexual problem, and j = other problems*)

9. XProblems with social relationships

10. Problems with activities of daily living

11. Problems with living condition

12. Problems with occupation and activities.

In this instrument each category is rated in the scale of 0 to 4 with zero for "no problem," 1 for “minor problem requiring no action,” 2 for “mild problem but definitely present,” 3 for “moderately severe problem,” and 4 for "severe to very severe problem". For the category #8 that lists 10 items of problems, one major problem is selected for each patient for rating on the same scale of 1 to 4. The scales and subscales of HoNOS [16,17] are HoNOS-Total for summed scores of items #1 to #10, HoNOS-Behavior for summed scores of items #1, #2, & #3, HoNOS-Impairment for summed scores of items #4 and #5, HoNOS-Symptom for summed scores of items #6, #7, & #8, and HoNOS-Social Functioning for summed scores of items #9 through #12. The HoNOS scale does not measure the level of risk, and neither the information regarding the risk nor the psychiatric diagnoses were available for this study. However, the level of risk can be inferred from the ratings on the categories of *overactive, aggressive behavior* and *non-accidental self injury*.

We constructed a clinical problem grouping from the data, as many patients had more than one problem rated on HoNOS. We categorize the HoNOS scores into two levels: “1” as no clinically significant problem (for the scores of 0 to 2), and “2” as clinically significant problem (for the scores 3 and 4) in order to identify co-occurrences of the problems. We also grouped the items of “*overactive/*aggressive”, “*problems with alcohol & drug abuse*”, “*cognitive problems*”, “*physical illness or disability problems*”, “*phobia*”, “*compulsive behaviours”,* “*dissociative*”, “*somatoform*”, “*eating disorder*”, and “*other problems*” as a consolidated category as “other problems” for this construction. This was done because there were only few patients on these items with the ratings of 3 or 4, except the item on “*physical illness or disability*” which was viewed to refer to non-mental health problem. The final instrument for the clinical problem type includes seven types labelled as specified in the following:

1. No Problem Type - No clinically significant problem

2. Stress only Type - One problem of stress only (anxiety, stress/tension, or insomnia)

3. Self-harm Type - Self-harm only or with other problems including depression

4. Psychosis Type - Psychotic problems only or with other problems including depression

5. Depression Type - Depression only or with other problems except self-harm and psychotic problems

6. Single Problem Type - One other problem only (Of those categorized as *other problems* in the recoding)

7. Miscellaneous Type - Two or more other problems

Because there was no case in which both psychosis and self-harm occurred together, it was possible to anchor psychosis and self-harm as the anchors independent of each other in constructing these types. However, as depression co-occurred with these problems, depression is used as an anchor for combinations involving neither psychosis nor self-harm.

In addition to HoNOS, patients were also rated on the Global Assessment of Functioning scales (GAF) both for symptoms (GAF-S) and functioning (GAF-F) at intake and discharge. GAF is a numeric scale (0 through 100) used by mental health clinicians and physicians to rate subjectively the social, occupational, and psychological functioning of adults (*e.g.*, how well or adaptively one is meeting various problems-in-living) [15,18]. Ten ranges of score specify the levels of symptom and functioning ranging from the highest level for no symptoms (GAF-S) and superior functioning in a wide range of activities (GAF-F) to the lowest level for persistent danger of severely hurting self or others (GAF-S) and persistent inability to maintain minimal personal hygiene (GAF-F).

### Data collection procedures

The team members of the CRHT team were trained to use the questionnaire including HoNOS and GAF at the time the team was established. The responsible team member for each patient at admission and discharge filled out the questionnaire. This data collection was done specifically for this research project. The researchers held quarterly meetings with the professional staff of the team in order to re-train their use of the registration form throughout the data collection period. The data were collected on all patients who went through the intake process for the team during the study period.

### Data analysis

The data were analyzed by the statistical software PASW for Windows version 17.0 for SPSS for descriptive statistics. When comparing groups the Student’s *t*-test or F statistics were used for continuous variables, and the Pearson´s chi-square test was used for categorical variables.

### Ethics

The Regional Medical Research Ethics Committee, Health Region II (South) of Norway and the Norwegian Social Science Data Services on behalf of The National Inspectorate approved this study.

## Results

### The sample – demographic, personal characteristics

The basic distributions in the demographic variables and the referral source are presented in Table 1. The study group consisted of more females than males, and a significantly lower proportion of the elderly over the age of 65 (6%) compared to the population in the DPS region (16%) and in Norway (15%). The distribution in the marital status is similar to the general adult population in Norway. The results show that only about one quarter of the total group had regular income, and more than one half were on disability/sick pay. Eight percent of the sample was on public social fund, of which the majority (81%) was those over the age of 65, most likely receiving government pension. A little less than half (40%) were living alone, and about one half of the young adults (ages 26-45) were responsible for childcare while only one third of the middle aged (ages 46-65) had child care responsibilities.

**Table 1:** Patient characteristics and referral source

**Variables**	**N (%)**
	**(Total N = 363)**
**Patient characteristics**	
**Gender** (Missing = 3)	
Male	127 (35.3)
Female	233 (64.7)
**Age** (Missing = 1)	
25 or younger	53 (14.6)
26-45	175 (48.3)
46-65	22 (23.4)
66 or older	20 (5.5)
Mean age: 41.31 years (SD = 14.219)	
**Marital status** (Missing = 9)	
Single	129 (36.4)
Married/Cohabit/Partner	151 (42.7)
Widowed	18 (5.1)
Divorced/Separated	56 (15.8)
**Ethnicity** (Missing = 1)	
Norwegian	309 (85.4)
Non-Norwegian	53 (14.6)
**Income source** (Missing = 29)	
Regular income	77 (23.1)
Dependent/student/Others	29 (8.7)
Sick/Disability pay	201 (60.2)
Public social fund	27 (8.1)
**Living Situation** (Missing = 19)	
Living alone	137 (39.8)
Living with someone	207 (60.2)
**Childcare responsibility** (Missing = 47)	
No child care	196 (62.0)
Part-time childcare	31 (9.8)
Full-time childcare	89 (28.2)
**Referral source** (Missing = 5)	
Self/family	141 (39.4)
GP	93 (26.0)
Emergency service	20 (5.6)
Psychiatric professional or service	37 (10.3)
Clinic/daycare/DPS	38 (10.6)
Others	29 (8.1)

### Referrals for admission

About three quarters of the patients (77%) were determined at the admission to be intakes to the CRHT team for emergency assistance to be provided within a 24 h period. The distribution in the referral sources is shown in Table 1. About 40% of the patients were referred to the team by self or family members. Patients and family in general would have known about the CRHT team in the community from their GPs or other healthcare providers, through their past experiences at mental health care service units, and from pubic media. An additional 26% were referred by primary care physicians. Twenty patients (6%) were referred by emergency units, suggesting that these patients were specifically referred for community-based crises care rather than being hospitalized by the emergency services. Twenty one percent of the patients were referred to the team by psychiatrists or psychiatric services such as mental health clinics or DSP daycare units. This means that half of the patients had an initial contact with healthcare professionals prior to their admission to the CRHT team. The majority (79%) were referred by those who were acquainted with the patients.

When the distributions in the referral sources by demographic variables (age, gender, marital status, ethnicity, living situation, income source, and child care responsibility) were examined, it was found that none of the variables was statistically significant in differences in the distributions. However there were some interesting differences as shown in Table 2. Female patients than male patients were more likely to be referred by self or family, while the middle-aged (46 to 65) among the age groups were most likely to refer themselves or by families. On the other hand, those married or widowed were more likely to refer themselves or be referred by their families compared to other marital status groups.

**Table 2:** Distribution in referral source by demographic characteristics

	**Referral source**					
	**Self/family**	**GP**	**Emer-gency**	**Mental health professional**	**Clinic/Daycare/DPS**	**Others**
**Gender**						
Male	42 (33.6)	36 (28.8)	11 (8.8)	13 (10.4)	13 (10.4)	10 (8.0)
Female	98 (42.6)	57 (24.8)	9 (3.9)	24 (10.4)	23 (10.0)	19 (8.3)
Statistics	*χ*2 = 5.618 (*df =* 5; *p >* .05)					
**Age**						
25 ≤	18 (34.6)	16 (0.8)	3 (5.8)	3 (5.8)	7 (13.5)	5 (9.6)
26-45	63 (36.3)	40 (23.3)	10 (5.8)	16 (9.3)	25 (14.5)	18 (10.5)
46-65	54 (47.4)	29 (25.4)	4 (3.5)	16 (14.0)	6 (5.3)	5 (4.4)
66 ≥	6 (30.3)	8 (40.0)	3 (15.0)	2 (10.0)	0 (−)	1 (5.0)
Statistics	*χ*2 = 23.812 (*df =*15; *p* = .068)					
**Marital Status**						
Single	47 (37.0)	35 (27.6)	13 (10.2)	13 (10.2)	10 (7.9)	9 (7.1)
Married	67 (45.9)	39 (26.2)	4 (2.7)	11 (7.4)	16 (10.7)	12 (8.1)
Widowed	8 (47.1)	6 (35.3)	0 (−)	2 (11.8)	0 (−)	1 (5.9)
separated	18 (32.1)	13 (23.2)	3 (5.4)	8 (14.3)	9 (16.1)	5 (8.9)
Statistics	*χ*2 = 17.763 (*df =*15; *p >* .05)					

*Significant at p < .05; **Significant at p < .01.

### Mental health problems at admission and previous mental health services

The distributions in mental health problems are presented in Table 3. Regarding the patients' status and experiences prior to admission to the team, nearly 80% of the patients were admitted to the team for the recurrence of an existing mental health problem or aggravation of a long-standing mental health problem, while only one fifth of the patients were admitted to the team for new or recent-onset mental health problems. During the 48 h prior to their intake, 38% of the patients were on psychiatric prescription medication, while 51% did not take any psychiatric drugs with the rest of the patients taking non-prescription drugs (11%).

**Table 3:** Distribution in status of mental health problems at intake, prior use of psychiatric services, use of psychiatric medication, selected HoNOS & scales, the clinical problem type, & GAF scales

**Variable**	**N (%)**
**Status of mental health problem at admission** (Missing = 55)	
New or recent episode	64 (20.8)
Recurrence after remission	76 (24.7)
Aggravation of chronic problem	168 (54.5)
**Prior psychiatric care** (Missing = 15)	
Yes	257 (73.9)
No	91 (26.1)
**Psychiatric medication use** (Missing = 23)	
Not taking any medication	172 (50.6)
Mostly prescription psychiatric drug(s)	129 (37.9)
Some prescription psychiatric drug(s)	15 (4.4)
Mostly non-prescription drug(s)	24 (7.1)
HoNOS – those having problems in selected categories (score of 3 or 4)*	
Self-harm	32 (8.8)
Substance abuse	29 (8.2)
Hallucinations & delusions	27 (7.5)
Depression	81 (22.4)
Anxiety	71 (19.6)
Stress/tension	67 (18.5)
Insomnia	27 (7.4)
**Clinical Problem Type** (Missing = 11)	
No Problem Type	109 (30.97)
Stress Only Type	73 (20.74)
Self-harm Type	29 (8.24)
Psychosis Type	26 (7.39)
Depression Type	62 (17.61)
Single Problem Type	31 (8.81)
Miscellaneous Type	22 (6.25)
**HoNOS Scales**	Mean (SD)
HoNOS-Total (Range: 1-26)	9.757 (4.679)
HoNOS-Symptom (Range: 0-11)	4.897 (1.791)
**GAF Scales**	Mean (SD)
GAF-Symptom	48.31 (10.513)
GAF-Functioning	48.11 (13.278)

*Many patients were in more than one category of HoNOS, and the base number for the percentages is the total sample (N = 363).

Although 74% had previous contacts with psychiatric services, about one half of the previous medical services received both during the 48 h (42%) and 3 months prior to the intake (56%) were with primary care physicians. During the 48 h prior to the intake one quarter of the patients (25%) had contacts with family and/or friends only, while about 18% made contacts with emergency units and 15% had contacts with psychiatrists or psychiatric, mental health services. During the 3 months prior to the intake, more than one half of the patients received services by primary care physicians only, and about 30% received services by the combination of primary care physicians and mental health care professionals. There were only few patients who did not receive medical services during this period (6.8%), suggesting a presence of mental health problems or other health problems prior to the crises in this population.

The distribution in the status of mental health problems at intake according to prior psychiatric care and psychiatric medication use in the 48 h preceding the intake were also significantly different among the groups as expected (Table 4). Those who received previous psychiatric services were more likely to have had long-standing mental health problems at intake, and most of those not on psychiatric medications were those with new or recent episodes of mental health problems. A large proportion (over 70%) of those who had new or recent mental health problems or who had recent recurrence of existing illness were seen only by primary care physicians during the previous 3 months period, while those who had long-standing mental health problems were seen mostly by psychiatric services in addition to primary care physicians. These suggest that the patients with long-standing mental health problems were receiving medical care prior to their admission to the services by the CRHT team. No significant relationship between the type of contacts made during the 48 h prior to the intake and the status of present mental health problems was found.

**Table 4:** Distribution in status of mental health problems at intake by prior psychiatric care, use of psychiatric medication, & services received 3 months prior to intake

**Services and medication use prior to intake**		**Status of mental health problems at intake**			**TOTAL N (%)**
		**New or recent episode**	**Recurrence after remission**	**Aggravation of chronic illness**	
**Prior Psychiatric Care** (Missing = 6)					
Yes	N (%)	18 (29.5)	62 (86.1)	153 (93.3)	233 (78.5)
No	N (%)	43 (70.5)	10 (13.9)	11 (6.7)	64 (21.5)
*χ*2 = 110.302** (*df* = 2; p < .001)					
**Psychiatric Medication Use** (Missing = 51)					
No medication taken	N (%)	46 (74.2)	37 (49.3)	51 (33.1)	134 (46.0)
Took mostly prescription drugs	N (%)	12 (19.4)	28 (37.3)	79 (51.3)	119 (40.9)
Took partly prescription drugs	N (%)	0 (−)	2 (2.7)	13 (8.4)	15 (5.2)
Took mostly non-prescription drugs	N (%)	4 (6.5)	8 (10.7)	11 (7.1)	23 (7.9)
*χ*2 = 36.104** (*df* = 6; p < .001)					
**Type of Combination of Services Received during 3Months Prior to Intake** (Missing = 63)					
Primary care physician only	N (%)	49 (76.6)	53 (70.7)	60 (37.3)	162 (54.0)
Primary care physician plus psych clinic, mental health teams, or community professionals	N (%)	5 (7.8)	16 (21.3)	80 (49.7)	101 (33.7)
Abuse team or psychiatric team only	N (%)	2 (3.1)	1 (1.3)	9 (5.6)	12 (4.0)
Other combinations without primary care physicians	N (%)	2 (3.1)	2 (2.7)	4 (2.5)	8 (2.7)
No service	N (%)	6 (9.4)	3 (4.0)	8 (5.0)	17 (5.7)
*χ*2 = 51.215** (*df* = 8; p < .001)					

*Significant at p < .05; **Significant at p < .01.

**Figure 1:**
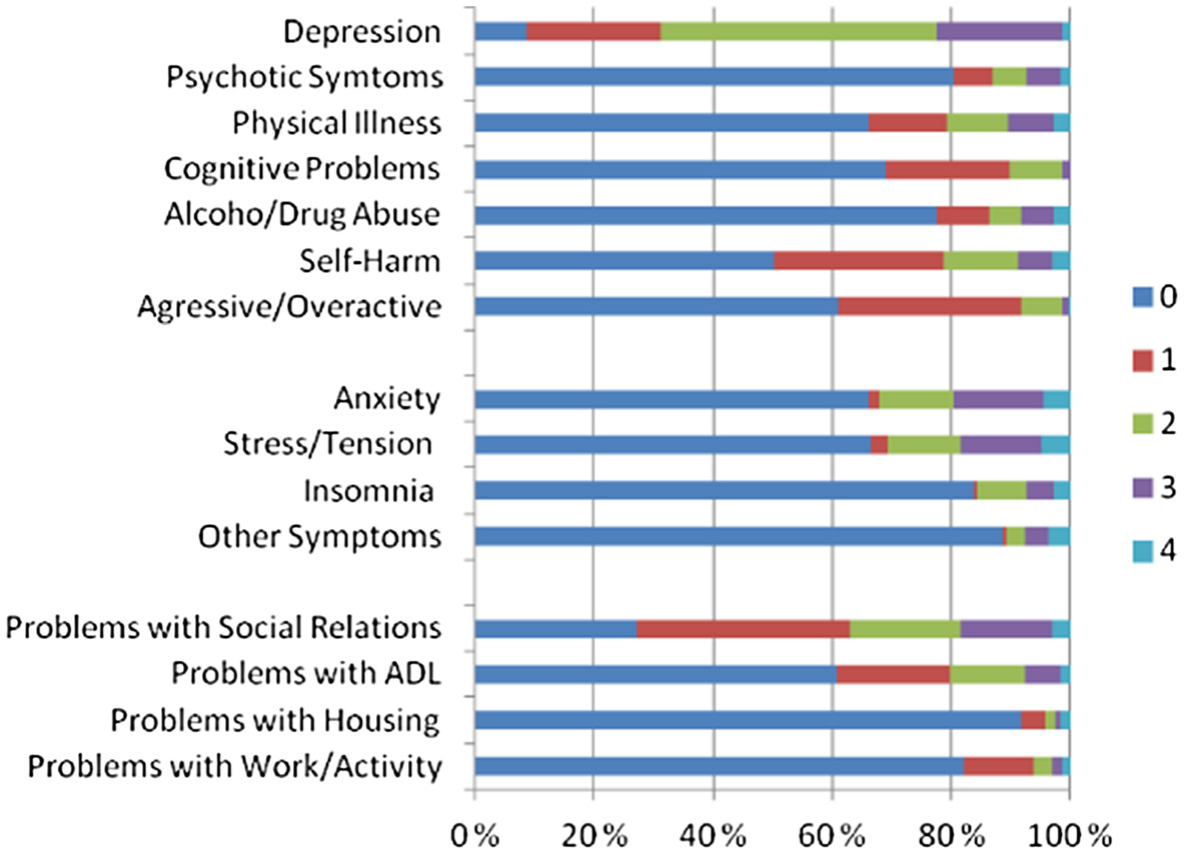
Distribution in the HoNOS categories at admission assessment††Most of the patients had more than one problem in these HoNOS categories.

The patients were assessed at admission regarding their mental health problems using HoNOS and GAF scales. Figure 1 and Table 3 show the distribution in HoNOS categories and problems at admission. In applying the rating of 3 on HoNOS items as the threshold for clinically serious mental health problems, the findings show that the most prevalent mental health problem in this group was depression (22%). In addition, about one fifth of the patients had anxiety (20%), stress/tension (19%), or the problem of insomnia (8%). Close to 10% of the patients had self-harm (9%), alcohol/drug abuse (8%), and psychotic problems (8%). There also were patients with problems related to physical illness/disability (11%). About one fifth of the patients had problems with social relations (19%) while a smaller proportion (8%) had problems with activities of daily living. The risk level to self and others, that may be inferred from the combined ratings on *aggressiveness* and *self-harm*, was not used in the study because there were only 4 patients with the score of 3 and 1 patient with the score of 4 on the “aggressiveness category” making the risk level mostly to be expressed by the scores on self-harm. The mean of 9.757 in the HoNOS-Total scale within the possible maximum score of 40, and the mean of 4.897 in the HoNOS-Symptom scale within the possible maximum score of 12 were at the low end of the scales, suggesting the mental health problems experienced by the patients to be in general not severe as measured by HoNOS.

**Figure 2:**
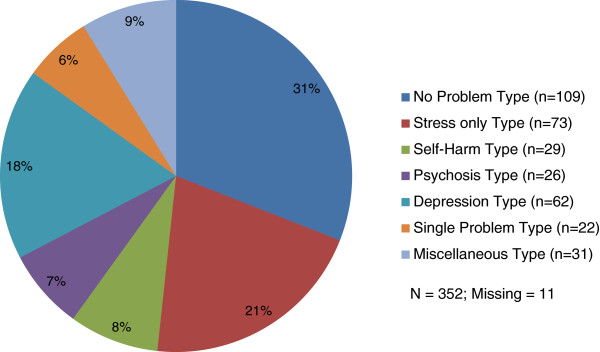
Number and percent in the clinical problem type at admission assessment.

The distribution in the clinical problem type is shown in Figure 2. By this grouping, nearly one third of the patients (31%) had no clinically significant problems, and an additional 21% had one stress response only. There were 8% in the self-harm type, 7% in the psychosis type, and 18% in the depression type as the major clinically significant problems.

Table 5 shows the mean scores on GAF-S and GAF-F for the three selected HoNOS categories, and by the clinical problem type. The mean values around 48 for both scales for the total group are at somewhat serious level both in terms of symptoms and functioning, somewhat contrary to the findings from HoNOS. As expected, the mean values of those with the selected HoNOS problems (self-harm, psychotic problems, and depression) were significantly lower, indicating poorer levels of functioning, than the means of those without the problems. Among the clinical problem types those with no clinically significant problems had the highest means in both scales, while the patients in the psychosis type had the lowest means in both scales. The correlations between the HoNOS-Total scale with GAF-S and GAF-F were -.592 and -.580 respectively significant at p = .01 level.

**Table 5:** Admission GAF-Symptom and GAF-Functioning scores by selected HoNOS categories and the clinical problem type at admission

		**GAF-Symptom**			**GAF-Functioning**		
		**Mean**	**SD**	**SE**	**Mean**	**SD**	**SE**
**Self-harm** (Missing = 2)							
	No (n = 329)	49.09	10.099	0.557	48.35	13.069	0.721
	Yes (n = 32)	39.75	10.106	1.786	45.22	14.466	2.557
*t*-test		t = 4.994**			t = 1.280		
**Psychotic Problems** (Missing =1)							
	No (n = 336)	49.52	9.541	0.521	49.21	12.823	0.700
	Yes (n = 26)	31.92	6.968	1.367	32.96	7.539	1.479
*t*-test		t = 9.209**			t = 9.934**		
**Depression** (Missing = 2)							
	No (n = 281)	49.90	10.008	0.597	50.36	12.834	0.766
	Yes (n = 80)	42.53	9.893	1.106	39.74	10.975	1.226
*t*-test		t = 5.830**			t = 7.342**		
**Clinical Problem Type**							
	No Problem Type	53.38	9.141	0.876	59.31	12.183	1.167
	Stress Type	50.49	7.892	0.924	49.67	11.079	1.297
	Self-harm Type	39.20	10.530	1.953	45.34	14.561	2.704
	Psychosis Type	31.96	7.109	1.422	32.24	6.716	1.343
	Depression Type	45.44	7.162	0.910	41.21	8.864	1.126
	Single Problem Type	53.00	11.320	2.033	51.29	13.118	2.356
	Miscellaneous Type	47.14	6.120	1.305	42.95	12.621	2.691
**Total** (N = 352; Missing = 11)		48.31	10.513	0.561	48.11	13.278	0.709
F statistics		F = 29.375** (*df* = 6,344)			F = 20.962** (*df* = 6, 344)		

*Significant at p < .05; **Significant at p < .01.

None of the demographic variables (age, gender, and marital status) was significantly associated with the HoNOS categories of self-harm, psychotic problems, and depression as well as with the clinical problem type. However, of those with self-harm 28% were 25 years or younger while of those without this problem only 13% were in this age group. Regarding the psychotic problem category, 22% of those with the problem were in the 46 to 65 age group compared to 32% of those without the problem. All sociodemographic variables except ethnicity were significantly different in relation to the status of mental health problems at intake as shown in Table 6. Males, those in the youngest age group, married/cohabiting, and those with regular or student/dependent income were more likely to have had new or recent episodes of mental health problems compared to others in the respective demographic groups.

**Table 6:** Status of mental health problems at intake by demographic variables

**Demographic/Personal variables**		**Status of mental health problems at intake**			**TOTAL**
		**New or recent episode**	**Recurrence after remission**	**Aggravation of chronic illness**	
**Gender** (Missing = 57)					
Male	N (%)	29 (45.3)	32 (42.7)	48 (28.7)	109 (35.6)
Female	N (%)	35 (54.7)	43 (57.3)	119 (71.3)	197 (64.4)
*χ*2 = 7.690* (*df* = 2; p = .021)					
**Marital Status** (Missing = 62)					
Single	N (%)	21 (32.8)	21 (28.4)	70 (42.9)	112 (37.2)
Married/Cohabit/Partner	N (%)	34 (53.1)	30 (40.5)	63 (38.7)	127 (42.2)
Widowed	N (%)	2 (3.1)	8 (10.8)	4 (2.5)	14 (4.7)
Divorced/Separated	N (%)	7 (10.9)	15 (20.3)	26 (16.0)	48 (15.9)
*χ*2 = 15.593* (*df* = 6; p = .016)					
**Age Group** (Missing = 55)					
25 or Younger	N (%)	17 (26.6)	8 (10.5)	21 (12.5)	46 (14.9)
26-45	N (%)	27 (42.2)	39 (51.3)	79 (47.0)	145 (47.1)
46-65	N (%)	19 (29.7)	19 (25.0)	62 (36.9)	100 (32.5)
66 or older	N (%)	1 (1.5)	10 (13.2)	6 (3.6)	17 (5.5)
*χ*2 = 21.546** (*df* =6; p = .001)					
**Income Source** (Missing = 81)					
Regular Income	N (%)	21 (34.4)	12 (17.1)	20 (13.2)	53 (18.8)
Dependent/Student/Others	N (%)	11 (18.0)	7 (10.0)	8 (5.3)	26 (9.2)
Sick/Disability Pay	N (%)	26 (42.6)	40 (57.1)	116 (76.8)	182 (64.5)
Public Social Fund	N (%)	3 (4.9)	11 (15.7)	7 (4.6)	21 (7.5)
*χ*2 = 35.424** (*df* = 6; p < .001)					
**Living Situation** (Missing = 72)					
Living alone	N (%)	13 (21.3)	32 (43.2)	76 (48.7)	121 (41.6)
Living with others	N (%)	48 (78.7)	42 (56.8)	80 (51.3)	170 (58.4)
*χ*2 = 13.673** (*df* = 2; p < .001)					

*Significant at p < .05; **Significant at p < .01.

### Referral source and mental health problems at admission

As shown in Table 7, the patients who had recurrence of existing problems were less likely to be referred by self/family and more likely by GPs than the other two groups, while those patients with long-standing mental health problems were more likely to be referred by psychiatric mental health professionals or clinics than the other two groups. The distributions in the referral source types according to three HoNOS categories and the clinical problem type are also shown in Table 7. Although none of these distributions are statistically significant suggesting a possibility of chance occurrences, there are some interesting differences. The patients with self-harm compared to those without self-harm seemed more likely to be either referred by self/family or by psychiatric mental health professionals or services. On the other hand, those patients with psychotic problems compared to those without the problem had a non-significant trend to be referred by psychiatric mental health professionals or services and less likely to be referred by self/family. There was no difference in the type of referral source between those with and without depression as the problem. In terms of the clinical problem type, the miscellaneous type had the highest proportion with self/family referral while the psychosis type had the lowest proportion with self/family referral. The patients in the depression type were most likely to be referred by self/family or by GPs (77% of the group). Thirty six percent of the patients in the self-harm type and 31% of the patients in the psychosis type were referred by psychiatric professionals or mental health services.

**Table 7:** Number and percent in referral source by selected HoNOS categories and the clinical problem type

	**Referral source**					
	**Self/family**	**GP**	**Emergency**	**Mental health professional**	**Clinic/Daycare/DPS**	**Others**
**Self-Harm** (Missing = 6)						
No	127 (30.9)	88 (27.0)	18 (5.5)	34 (10.4)	31 (9.5)	28 (8.6)
Yes	13 (41.9)	5 (16.1)	2 (6.5)	3 (9.7)	7 (22.6)	1 (3.2)
*χ*2 = 6.952 (*df =*5; *p >* .05)						
**Psychotic Problems** (Missing = 5)						
No	135 (40.8)	85 (25.7)	18 (5.4)	32 (9.7)	35 (10.6)	26 (7.9)
Yes	6 (22.2)	8 (29.6)	2 (7.4)	5 (18.5)	3 (11.1)	3 (11.1)
*χ*2 = 4.733 (*df =*5; *p >* .05)						
**Depression** (Missing = 6)						
No	106 (38.4)	69 (25.0)	18 (6.5)	29 (10.5)	30 (10.9)	24 (8.7)
Yes	34 (42.0)	24 (29.6)	2 (2.5)	8 (9.9)	8 (9.9)	5 (6.2)
*χ*2 = 3.127 (*df =*5; *p >* .05)						
**The Clinical Problem Type** (Missing = 16)						
No Problem Type	40 (37.4)	29 (27.1)	5 (4.7)	13 (12.1)	10 (9.3)	10 (9.3)
Stress Only Type	29 (40.8)	18 (25.4)	3 (4.2)	5 (7.0)	9 (12.7)	7 (9.9)
Self-harm Type	11 (39.3)	4 (14.3)	2 (7.1)	3 (10.7)	7 (25.0)	1 (3.6)
Psychosis Type	6 (23.1)	7 (26.9)	2 (7.7)	5 (19.2)	3 (11.5)	3 (11.5)
Depression Type	27 (43.5)	21 (33.9)	2 (3.2)	5 (8.1)	3 (4.8)	4 (6.5)
Single Problem Type	13 (41.9)	9 (29.0)	2 (6.5)	3 (9.7)	1 (3.2)	3 (9.7)
Miscellaneous Type	10 (45.5)	5 (22.7)	2 (9.1)	3 (3.6)	2 (9.1)	0 (−)
*χ*2 = 24.462 (*df =*30 *p >* .05)						
**Status of mental health problems at intake** (Missing = 55)						
New or recent onset	27 (42.9)	15 (23.8)	3 (4.8)	4 (6.3)	9 (14.3)	5 (7.9)
Recurrence after remission	23 (31.1)	27 (36.5)	4 (5.4)	4 (5.4)	5 (6.8)	11 (14.9)
Aggravation of chronic problem	69 (41.6)	33 (19.9)	11 (6.6)	23 (13.9)	19 (11.4)	11 (6.6)
*χ*2 = 18.340* (*df =*10 *p <* .05)						

*Significant at p < .05; **Significant at p < .01.

## Discussion

The results of this longitudinal survey of a CRHT team in Norway have provided insights into the demographic backgrounds of the patients, how they were referred to the team, and what problems were identified at admission. Although our findings confirmed the general characteristics of patients served by CRHT teams reported for 8 teams in Norway [15], there are key findings in this study which provide more detailed picture regarding the characteristics of patients at intake. The discussion addresses the key issues from the findings regarding (a) the patient characteristics, (b) a lack of representation by older adults for the service, (c) the referral patterns, and (d) the nature of mental health problems. The findings of this study needs to be interpreted and understood from the Norwegian context of its healthcare system, which operates within the national health insurance framework. This means that cost considerations from the individual perspective in seeking medical and health services are not relevant in Norway while they are critical in privatized systems such as in the US. As the Norwegian context is comparable to the UK, Australian, and Canadian settings, it is possible to make comparisons of our data with those from these countries. The findings also have to be understood in the context in which the CRHT was functioning: (a) the CRHT team had been established about six months prior to the start of data collection, suggesting that the team was still in the learning mode, (b) its operating hours were limited to the day and evening hours on weekdays and day hours on weekends, which means that it was not able to meet the requirement for 24/7 availability for services, (c) there was no staff turnover during the data collection, and (d) it did not have the official gate-keeping authority for psychiatric hospitalization for the DPS, although it performed evaluations of referrals that resulted in hospitalization.

Our findings on personal demographic characteristics are similar to the findings for eight CRHT teams in Norway by Hasselberg, Gråwe, Johnson, & Rudd [15]. We found that the patients of the CRHT team were likely to be those on public financial support mostly due to disability and illness, as was found in the study by Hasselberg *et al.*[15] reporting only one-quarter of the patients to be in paid employment. These suggest that most of the users of CRHT teams are likely to be those who have long-term mental health problems by which they probably become designated for disability pay. Nearly four fifth of the patients also had long-standing mental health problems or recurrence of existing mental health problems at the time of admission and had been receiving healthcare for them. However, the patients in general did not have severe mental health problems at admission to the CRHT team, although they could have been in crises. These findings suggest that mental health crises may have specific meanings, not necessarily related to a high level of vulnerability to hospitalization, especially to patients who were self referrals to the CRHT team. It is also possible that those referrals to the team by GPs and community mental health professionals may be the results of differentiation by them regarding mental health crises in terms of those requiring hospitalization and those requiring the service of a CRHT team. This seems to suggest that there are two levels of acute mental health crises, one requiring hospitalization and the other resolvable through community-based care. Since the CRHT team did not have an official authority for the gatekeeping role for acute hospitalization in the DPS, it is not possible to determine whether or not this was the process in place. The findings suggest that individuals with long-term mental health problems may often be in need of crisis care for which CRHT teams can play a critical role with their resolution. Whether or not the CRHT played any role in preventing hospitalization is not known in our data. In view of the mandate for the establishment of community mental health services, especially for the functional teams such as CRHT, to prevent hospitalization of people with long-standing mental health problems, these findings require further investigation, especially to examine the conjecture that the availability of CRHT teams may avert hospitalization of patients with long-term mental health problems, by making it possible for them to receive emergency crisis care within the 24 h window in the community, as suggested by Johnson and colleagues [13,19].

### Where are older adults with mental health problems?

We found in this study that this patient-group was represented by more females and those between the ages of 26 and 65 years. Older adults in the ages over 65 were under-represented in the team (6%) compared to the general population in Norway (16%), indicating that older adults were not likely to be referred to CRHT services. Although there are health service units in the community dedicated to the health care of older adults in Norway such as geriatric community health services, day-care for the elderly, and psycho-geriatric units in hospitals, older adults with mental health problems receive services in the same manner as adults in general especially in relation to mental health crises.

Although it is not possible to determine the exact "local" reasons for this low representation of older adults in the study group, the reasons for this finding may be considered as multiple and interconnected. For example, older adults may experience mental health crises differently, they may be able to deal with crises with strategies learnt throughout their lives, they may not be as aware of the availability of the program as well as younger adults, or they may be more reluctant to seek services for mental health problems. The absence of older adults in community mental health care in Norway is also found in the evaluation of the national action plan [20]. Our findings are also in line with the results of the study by Hasselberg *et al.*[15] of 8 crisis resolution teams in Norway, as they found that most patients were between 20–50 years of age. Bogner *et al.*[21] also reported that older adults aged 60 years and older of their sample of over 1,000 community-dwelling adults in Baltimore were less likely to consult with mental health specialists and more likely to receive mental health care from primary care physicians. Karlin and Norris [22] also found a lower rate of the use of public mental health services by older adults compared to the younger groups.

In general older adults are vulnerable to mental health problems because of (a) the decline in physical and psychological robustness in older age, (b) the shrinking social support system, and (c) changes in personal situations such as retirement, the loss of a spouse or relocation. Studies from different countries for example, [23-25] have shown the prevalence rates of depression among older adults to be between 10 to 20%. Depression represents the major portion of mental health problems in older adults [23], and depression in older adults is associated with frailty [23], increased dependence in ADL [26], and physical comorbidity [27]. Furthermore, Burroughs *et al.*[28] suggested that both primary care practitioners and patients view depression in older adults as justifiable and that older adult patients specifically consider depression with passivity, limited expectations of treatment, and not necessarily legitimate reason for seeking medical help. As there is a paucity of studies looking into the reasons for the lack of representation by older adults for this type of service, it is critical to gain an understanding regarding this issue in order to have insights about the nature of mental health problems experienced by this age group and the reasons for low utilization of community mental health services by older adults.

### Patient referrals

Referral to the team by self or family members in large numbers suggests that patients or their families were able to apply certain criteria to make decisions about their situations of mental health crises. It is likely that since most patients had long-term mental health problems they or their families were able to make decisions regarding when to seek crisis care. The finding is important also in another sense in that some of CRHT services only allow referrals by professionals [2], in which cases patients would have to go through an additional step in health services in order to be referred to CRHT teams.

Our findings also suggest an important role played by primary care physicians in referring patients to CRHT teams. It is possible that many people consider their primary care physicians as initial contacts for most of medical care within the healthcare system. This also has implications regarding the low representation of older adults for this type of services discussed earlier, as primary care physicians may play a gate-keeping role for mental health care for older adults especially if they were guided by the attitude regarding depression in older adults as 'justifiable' and not requiring psychiatric treatment.

In addition, the finding that about half of the patients were referred to the CRHT team by healthcare professionals and services including mental healthcare and emergency services suggests that these referring professionals and services made judgments regarding the appropriateness of the services by the CRHT team for these patients. However, it is not clear from the data whether the establishment of the CRHT was filling a need that existed in the community for mental health crises care or it was an additional service that became available re-distributing the services for mental health in the community. Since the data for this paper were only from the patients who were admitted to the CRHT team, it is not possible to compare the referral sources of those admitted to the team and those who were referred to the team but not admitted to the team.

### The nature of mental health problems

The findings that most of the patients had received mental health care previously and had existing mental health problems suggest that mental health crises are intrinsically related to existing mental health problems. However, the long-term mental health problems these patients had may not be severe psychiatric disorders such as schizophrenia and schizoaffective disorders. In line with the findings by Hasselberg *et al.*[15], the level of mental health problems the patients experienced at admission was not severe clinically with about one third of the patients having no clinically significant problems and one fifth with only stress responses, along with additional one fifth with depression and only 15% with self-harm and psychotic problems. The study of a crisis resolution and home treatment team in Edinburgh by Barker *et al.*[14] found 17% of their sample with the diagnosis of schizophrenia/schizoaffective disorders and 25% with depression on ICD-10, while somewhat differently Johnson *et al.*[13] in a quasi-experimental study of CRT found 25% of the sample to have the diagnoses of schizophrenia/schizoaffective disorders, 55% with psychotic symptoms, 14% with elevated mood, and 59% with depressive symptoms. The proportions of persons with depression appear to be in the range of 25 to 59% in these studies in line with our finding, suggesting the magnitude of depression in relation to mental health crises. The significant differences are in the prevalence of psychosis reported in different studies. Such disparities in the diagnostic make-up of patients seeking crisis care may be due to the instruments used for the assessment, *i.e.*, HoNOS *versus* ICD-10, to the different service configurations in the community for mental health care, or because of the different sampling bases for the studies. The low number of patients with psychosis in this study may be due to the possibility that patients with long-term psychosis may be aligned with community psychosis/rehabilitation teams and receive crisis care as well as the routine care from this type of teams available at the DPS level in Norway.

However, since more than two thirds of the patients in our study were judged to be in need of emergency assistance at admission and more than half of the patients were referred by healthcare professionals including 21% by mental health professionals and services, it seems that there apparently were needs for services by the CRHT team for these patients. It is possible that these professionals held a view of mental health crises which can be addressed successfully by CRHT services, although it is possible that the thresholds for referrals for crisis care by mental health professionals and by primary care physicians may be different in general, and that patients with more serious mental health crises may have been referred to inpatient psychiatric emergency units bypassing CRHT teams completely. There were no data available in this study regarding the exact nature of crises that brought the patients to this service. However, the pattern of the clinical problem types extracted for this group of patients indicate that mental health crises may not be clinically definable, and may culminate from complex problems.

In addition to the clinical problems such as stress responses, depression, and self-harm, problems with social relations and daily activities were prevalent in the patients, suggesting that mental health crises are not simply associated with clinical symptoms but also with problems of daily life. Of the 18 CRHT teams in Wales surveyed by Jones and Jordan [29] only six teams accepted patients with problems with social relations or daily activities, suggesting that by themselves these may not be considered as mental health crises by some CRHT teams. Jones and Jordan [29] also reported that most of the teams accepted patients with psychosis, affective disorders, substance misuse disorders, personality disorders, and anxiety disorders. Although the clinical problem types constructed in our data are somewhat different from these types, our results correspond with the acceptance of various mental health problems represented by such disorders for the team's services. While the exact nature of mental health crises is unknown, CRHT teams in general respond to crises stemming from many different types of mental health problems.

Given the characteristics of the patients who received services from the CRHT team, there is a need for further research to gain understandings about the nature of mental health crises that bring patients to the attention of CRHT services and their relationships with psychiatric diagnoses. One limitation in the study regarding mental health problems is the lack of risk measures. Since mental health crises have implications for risks to self and others, it is critical to have a sensitive instrument to measure the risk. With a sensitive risk measurement it may be possible to capture the nature of mental health crises that are appropriate for CRHT teams, even if the overall level of clinical problems is not intense. There also is a need for further research regarding the use of assessment tools such as HoNOS in relation to mental health crises and psychiatric diagnoses.

It is neither clear nor possible in this study to judge how many of these patients would have been hospitalized without the services by the CRHT team or whether they would have received similar services from other sectors of mental health care in the community. Especially since the CRHT team did not have an official gate-keeping role for hospitalization within the DPS, the impact of CRHT on hospitalization rate is difficult to assess. A further step in our project could be to obtain psychiatric hospitalization data including diagnoses retrospectively in the DPS for the study period in order to compare patient characteristics of the study group to the inpatient group. This could provide a deeper understanding regarding the role CRHT teams play in managing mental health crisis in the community. There is a need to study the reasons for referral from the perspectives of self/family and healthcare professionals in order to gain a greater understanding of the meanings of mental health crises and the role of CRHT services.

The major clinical implication of the results of this study is in relation to the role of professional services by CRHT teams. The clinical characteristics of patients seeking services by CRHT teams may influence the types of services provided to them. Since the severity level of mental health problems is not intense in this population, the modes of crises care may need to be reframed by how mental health crises are viewed by patients, healthcare professionals, and professionals within CRHT teams.

### Methodological considerations

There are several shortcomings related to this study. The data on HoNOS and GAF for the level of mental health problems at admission and at discharge were based on the team members´ subjective assessments. The team members were trained on the use of these instruments several times during the course of the study and there was no staff turnover in the team during the data collection, suggesting a high level of standardization in data collection with these instruments in the study. However, there are possibilities for registration or recall bias, for which reliability or validity testing was not done in this study.

This being a study of one CRHT team makes generalization of the findings difficult, although the results provide a basis for gaining insights regarding the workings of CRHT teams. Furthermore, the setting of this study is Norway, which has a specific healthcare system including mental health care. Therefore, the referral patterns found in the study may be very specific to Norway.

The assessment instruments used in the data (*i.e.*, HoNOS and GAF) are general and do not provide information regarding the nature of mental health crises. There is a need for a better assessment tool regarding mental health crises.

## Conclusions

CRHT has been established as a somewhat new functioning unit within the mental health care system with a specific mandate to treat people in mental health crises in the community. Our findings of one CRHT team over a period of 18 months provide insights as to the general profile of patients seeking services by such teams. An underrepresentation of older adults for this CRHT team and the corresponding literature suggest a need to examine further the reasons for and the consequences of under-utilization of CRHT as well as other types of mental health services by older adults. As the CRHT team served mostly patients with existing mental health problems and the level of severity of mental health problems was not intense, there is a need to re-examine the role of functional teams such as CRHT teams in relation to general community-based mental health care services. Since our findings indicate that stress responses and depression are critical components of mental health crises, it is important to have future research focusing specifically on the nature of mental health crises and the structure of morbidity in order to gain knowledge regarding the effectiveness of CRHT services in resolving mental health crises.

## Competing interests

The authors declare that they have no competing interests.

## Authors’ contributions

All authors were actively involved in the research project and contributed to all aspects in the preparation of the manuscript. All authors read and approved the final manuscript.
